# Implementation of the health promoting school program in Poland: results from a national cross-sectional study

**DOI:** 10.1186/s12889-026-28984-9

**Published:** 2026-08-17

**Authors:** Karina Leksy, Grzegorz Gawron, Kevin Dadaczynski

**Affiliations:** 1 Institute of Pedagogy, Department of Social Science, University of Silesia in Katowice, Katowice, Poland; 2 Institute of Sociology, Department of Social Science, University of Silesia in Katowice, Katowice, Poland; 3 Faculty of Human Science, Department Sports and Health Sciences, University of Potsdam, Potsdam, Germany; 4 Center for Applied Health Science, Leuphana University , Lueneburg, Germany

**Keywords:** Health promoting schools, Implementation research, Fidelity, Adaptation, Integration, Whole-school approach, Poland

## Abstract

**Background:**

The Health Promoting School (HPS) approach is widely recognised as a promising avenue for supporting the health and wellbeing of pupils, teachers and non-teaching staff at the intersection of schools and communities. Although Poland has a long tradition of establishing a national HPS Network, empirical evidence on the level and determinants of implementation remains limited. This study aims to assess the level of implementation of health promotion in Polish schools belonging to the national HPS network and to examine differences in terms of school type, National HPS Certification, and length of network membership.

**Methods:**

A cross-sectional survey was conducted between March and June 2023 among health promotion coordinators from public Polish schools participating in the HPS network (*N* = 500, completed questionnaires = 426, i.e., 85.2%). The HPS Implementation Questionnaire was used to measure seven dimensions of implementation (e.g., fidelity, adaptation, integration). Differences between groups were analysed using t-tests and one-way ANOVA.

**Results:**

Overall implementation of the HPS approach was moderate-to-high (*M* = 3.05, *SD* = 0.62). The highest scores were found for *Integration* (*M* = 3.53, *SD* = 0.74) and *Adaptation* (*M* = 3.40, *SD* = 0.88), while *Adherence* (*M* = 2.09, *SD* = 0.70) and *Participant responsiveness* (*M* = 2.71, *SD* = 0.84) scored lowest. Group comparisons revealed higher implementation levels across most dimensions for primary schools, schools holding a National HPS Certificate and those with longer network members.

**Conclusions:**

The findings indicate that sustained participation in the HPS network is associated with higher implementation of school health promotion. Schools holding the National HPS Certificate scored higher on all implementation dimensions, a pattern consistent with the rigorous requirements of the certification process. The results highlight the importance of long-term capacity building, supportive structures, and contextual adaptation in achieving effective and sustainable HPS.

## Background

For many years, initiatives and strategies aimed at improving pupils’ health have been implemented in schools worldwide. Within school health promotion the ‘whole-school approach’ is considered the most comprehensive as it *‘goes beyond the learning and teaching in the classroom to pervade all aspects of the life of a school*’ [1]. The overarching goal is to provide support to students in relation to their learning, behaviour and well-being, both within and beyond the classroom environment [2, 3]. Back in 1995, the World Health Organisation (WHO) launched a Global School Health Initiative, which describes a Health Promoting School as a school that is constantly strengthening its capacity as a healthy setting for living, learning and working. As part of the initiative ‘Making Every School a Health Promoting School’, launched jointly by WHO and UNESCO [1], eight global standards were developed that address different levels (school curriculum and school physical environment) and also cover management-related and school-government aspects (e.g., policies and resources, leadership). Despite differences in the orientation of whole-school approaches to health, commonalities can be identified in that they address health at the curricular level, shape the physical and social environment, and initiate networking activities outside of school, such as with parents and the community [4, 5]. Since its inception, numerous attempts have been made to support schools in implementing the Health Promoting School (HPS) approach. Although evidence is mixed and sometimes lacking [5, 37, 38] there is broad consensus that its introduction and long-term implementation are crucial for health, well-being and educational success [1, 4, 6]. The call to ‘*make every school a health-promoting school*’ is recognized as an important driving force behind current global progress for which guidelines for the adaptation and implementation of global standards for HPS have been developed. Central to effective implementation are distributed models of school governance and leadership, strong partnerships between schools and local communities, and curricula that explicitly and implicitly promote physical, social, and psychological health through inclusive and evidence-informed pedagogy. This further underscores the importance of supportive social–emotional climates, safe and accessible physical environments, and comprehensive school-based or school-linked health services, positioning health and well-being as foundational conditions for educational quality, equity and long-term sustainability of the HPS model [1, 6].

As argued by Samdal and Rowling [7], HPS implementation can be conceptualised as a dynamic, continuous process involving planning, action, reflection and improvement [7, 8]. The successful implementation of such initiatives depends on several key factors, including, but not limited to, the presence of a strong school leadership [9–12] and a conducive organisational culture, active engagement of students [13, 14], teachers, parents [15], and community stakeholders [6, 16–18], effective collaboration between the education and health sectors, the adaptation of programmes to local contexts [19, 20], and ongoing monitoring and evaluation [7, 13]. It also depends on community involvement (e.g., funding, support) [21, 22], socio-cultural and historical background [23] and the national policy (e.g., political and financial support) [24–26].

Given its complexity, the implementation of the HPS approach varies considerably between and within countries [3, 27]. While some countries have established a formal national policy on HPS, others have incorporated aspects of school health into broader national education policies while others have no such policies in place [28]. Research has shown that some schools use the HPS approach on a daily basis, while others do not, or only to a limited extent [27, 28]. Moreover, differences between types of schools have been reported with primary schools achieving a higher implementation level compared to other schools, mainly secondary and secondary vocational schools [29, 30]. Vilaça et al. [28] utilised six key components of a whole school approach to health to assess the extent of HPS implementation across European countries: (1) Healthy school policies, (2) School physical environment, (3) School social environment, (4) Individual health skills and action competencies, (5) Community links, (6) Health services. In addition, the authors estimated the percentage of schools in each country that had adopted the HPS approach. Based on their estimations, a minimum of 36,000 schools in Europe and Central Asia implemented activities in accordance with the HPS principles.

To sum up, interventions based on the HPS approach are complex, in that they comprise multiple components, are highly context-sensitive and depend heavily on the behaviour of both participants and implementers; their implementation must be seen as a non-linear and highly dynamic process [31–33]. Moreover, despite the evidence demonstrating positive effects of HPS, it is difficult to determine which intervention components and implementation strategies contribute most to their effectiveness [34–39].

## The health promoting school programme and its implementation in Poland

After the World Health Organization introduced the HPS concept in the late 1980s, its practical application was tested between 1992 and 1995 as part of a pilot project implemented in the Czech Republic, Poland, Slovakia and Hungary. In Poland, the initiative to establish HPS was launched as early as 1991.

The Polish HPS Network, coordinated by the Centre of Education Development (ORE) in Warsaw (a subordinate entity of the Ministry of National Education), has been involved since the project’s inception. The Network’s current activities primarily include disseminating the HPS concept in Poland, organising and facilitating workshops and seminars for school coordinators, supporting regional coordinators, promoting school health initiatives, and facilitating the exchange of good practices between schools. In addition, it supports the accreditation of HPS certifications at regional and national level and disseminates relevant materials and best practices. Poland has developed a unique national framework for healthy schools, grounded in the principles of the HPS framework. The framework is based on six key elements, as follows: (1) a comprehensive understanding of the concept, (2) extensive community involvement, (3) the presence of an active school health promotion team, (4) systematic planning and evaluation, (5) the integration of health promotion into the school´s core objectives, and (6) the promotion of close collaboration with the local community. Moreover, the Polish HPS programme is guided by four national standards [40] which address:

- School structures that support meaningful participation and long-term health promotion,
- A positive social climate for the whole school community,
- High-quality health education for students and staff,
- Healthy and supportive learning and working conditions, including cooperation with parents.

The implementation of the HPS programme is based on an assessment of the health needs of the school community. The school health promotion coordinator and HPS team are responsible for developing a 3-year health promotion programme for the school. This programme is based on the initial diagnosis and the knowledge and skills acquired during the training. The implementation and evaluation of the HPS programme falls within the remit of school health promotion (HP) coordinators (mostly full-time teachers, or, less frequently, the principal or vice principal). The coordinators adopt a dual role, acting both as leaders and as agents of change. They are responsible for the initiation and facilitation of the implementation process, and the creation of conditions conducive to its success [40].

The coordinator’s work is supported by a school health promotion team comprising three to five members, including representatives from all segments of the school community (e.g., other teachers, school nurse, students, parents and members of the local government or other organisations) [40]. HP coordinators are supported by regional and national HPS coordinators, who offer regular training and ongoing supervision, with a particular emphasis on the early stages of programme development.

By 2024, approximately 3,000 schools had been involved in this programme [41]. In the Polish HPS network a certification path has been developed. Schools that meet defined criteria, can join the Regional Network of Health-Promoting Schools and obtain a Regional Health-Promoting School certificate for three years. Following a three-year period, interested schools are permitted to submit an application for renewing the regional certificate, or to expand the scope of the health needs assessment and submit an application for a national certificate. Receiving a national certificate requires at least three years of membership in the provincial network, submitting the required documentation along with a recommendation from the provincial coordinator, demonstrating their own best practices, and disseminating information about the school’s health promotion activities. The acquisition of a national certificate is associated with a more thorough and comprehensive assessment of health needs in schools and a higher level of implementation of HP programmes. Despite the long tradition of the HPS approach in Poland and its many years of practical experience, there is a lack of empirical evidence on its implementation in Polish schools and possible differences. Therefore, this paper aims to assess the level of implementation in Polish schools belonging to the HPS network. The following research questions guided this research:

1. What is the implementation status of HPS in Polish schools that belong to a HPS network?
2. Are there differences in HPS implementation according to school type, and membership length in the Polish HPS network?
3. Do schools holding a National HPS Certificate score higher on implementation dimensions compared to schools without a certificate?

In this study, it is hypothesised that HPS characteristics (e.g. membership length in the HPS network and possession of the National HPS Certificate) may differentiate the overall level of implementation as well as implementation within subdimensions.

Vennegoor et al. [42] and Adamowitsch et al. [30] suggested that effective communication and active engagement are easier to achieve in primary schools. It is a commonly held view that primary school teachers typically devote most of their day to pedagogical activities and spend a lot of time with their pupils. This suggests that they may possess a greater sense of ownership for pupils’ health and be more involved in HPS activities. Secondary school teachers tend to have a different approach, distinguished by greater fragmentation and closely aligned with the subjects taught [30]. Finally, we examine whether schools holding the National HPS Certificate score higher on implementation dimensions compared to non-certified schools. In the Polish system, national certification is awarded to schools that demonstrate sustained, high-quality implementation over at least three years, including systematic planning, comprehensive health needs assessment, and dissemination of good practices. Given these requirements, we expect certified schools to show higher implementation scores. However, because certification is granted based on demonstrated implementation quality, any observed differences reflect the alignment between certification criteria and implementation dimensions.

## Methods

### Study design and data collection

The research was conducted between March and June 2023 among public schools belonging to the HPS network in Poland with the HPS coordinators as the primary target group [43]. At that time there were 2,990 schools in the Network [44]. The survey was realised in cooperation with the National HPS Coordination Centre (*Centre* for *Education Development* in Warsaw), which supported in disseminating the questionnaire among 16 HPS regional coordinators. Regional coordinators then sent the request to participate in the study to schools in the HPS Network in their region, with two reminders (at monthly intervals) to increase responsiveness and participation rate. The research was conducted using the online research tool LimeSurvey. Participants were informed about the study’s purpose and relevance, and active consent had to be given before starting. Completing the questionnaire took approximately 30 min. In total, 500 coordinators decided to participate in the study, of which 426 completed the entire questionnaire (85.2%). Hence, 16.7% of schools in the Polish HPS Network participated in the study (426 of 2,990 schools). Due to a lack of data in the HPS Network Poland, it is not possible to compare the distribution of the sample by school type. When compared to the overall population of all schools, primary schools are slightly more represented in the present sample (70.6% versus 67.0%). However, data on other population-level characteristics (e.g., regional distribution, certification status across the full network) were not available for systematic comparison.

### Demographic and school characteristics

Participants were asked to provide additional sociodemographic and school-related information as independent variables: type of school (primary/secondary); membership length in the HPS network (< 5 years; 5–10 years; >10 years); possession of the National HPS Certificate (yes/no); sex (male/female); age range in years (≤ 29; 30–39; 40–49; 50–59; ≥60) and years of professional experience (< 5; 5–15; 16–25; 26–35; >35).

Given the fact that the HPS network has been implemented in Poland for a long time, the present study used three periods in order to operationalise the membership length in the network (Table 1). This division is based on the hypothesis that educational institutions with a longer membership and greater experience will demonstrate higher levels of programme implementation.

**Table 1:** Membership length in the Polish HPS network

Years of participation	Description
up to 5 years	The initial phase of school membership of the network, including a preparatory period (minimum 6 months), a candidate phase (1 year), and initial membership in the Regional Health Promoting Schools network (3 years)
5–10 years	The second phase of network membership, during which schools can apply for • a 5-year National Health Promoting School Certificate or • a renewal 3-year Regional Health Promoting School certificate
over 10 years	Schools with extensive experience in implementing the Health Promoting School programme

## Measurements

### HPS implementation

The HPS Implementation Questionnaire, a tool developed by Maastricht University and the Netherlands Organisation for Applied Scientific Research, was used for this study [45].

The HPS Implementation Questionnaire was adapted for use in the Polish context following established guidelines for cross-cultural instrument adaptation [46]. The process involved: (1) forward translation into Polish by a bilingual researcher with expertise in health promotion, (2) review and refinement by national and regional HPS coordinators to ensure conceptual equivalence and contextual appropriateness, and (3) back-translation into English by a native English speaker unfamiliar with the original instrument. Discrepancies were resolved through consensus discussion among the research team and HPS coordinators. The questionnaire covers seven dimensions of implementation with a total of 28 items that could be rated on a five-point Likert scale ranging from 0 (‘definitely not’) to 4 (‘definitely’):

1. *Adherence* (i.e., the extent to which programme components are delivered in accordance with the underlying theoretical guidelines, 12 items),
2. *Dose* (i.e., the amount of programme content received by the target group, 5 items),
3. *Participant responsiveness* (the degree to which the target group is engaged with the programme, 1 item containing 9 examples),
4. *Quality of delivery* (i.e., how the programme is delivered, 5 items),
5. *Programme differentiation* (i.e., the extent to which the programme contains unique elements to achieve effective outcomes, 1 item),
6. * Adaptation* (i.e., the extent to which changes have been made to the programme, 1 item),
7. * Integration* (i.e., the extent to which the programme is integrated into the school system, 3 items).

The internal consistency of the Polish version was assessed using Cronbach’s alpha, yielding excellent reliability for the overall scale (α = 0.921) and acceptable to excellent reliability for the subscales (α = 0.853–0.921).

### Data analysis

Mean values for each subscale are presented on a scale from 0 to 4, with 4 representing the highest level of implementation. Student’s t-test (or Welch’s in case Levene’s test was significant at *p* = 0.05) was used to assess differences in HPS implementation between school types (primary vs. secondary) and between schools with and without National Certificate of HPS. Cohen’s d was used to estimate the effect size, which is classified as small (*d* ≃ 0.2), medium (*d* ≃ 0.5) or large (*d* ≥ 0.8) [47]. To examine whether the HPS implementation differs significantly depending on three independent groups of membership length in the national HPS network (< 5 years, 5 to 10 years, > 10 years), a one-factor analysis of variance (ANOVA) with a post-hoc Bonferroni test was performed. Eta squared (η²) was calculated as a measure of effect size for the ANOVA using the following interpretations (small < 0.06, medium < 0.14, large ≥ 0.14). The level of statistical significance was set at *p* < 0.05. Data analysis was conducted using IBM Statistical Package for Social Science (SPSS) Version 29.0 for Windows.

## Results

### Sample

A total of 500 HPS coordinators participated in the study, of whom 426 (85.2%) completed the questionnaire. The sample was primarily composed of HPS coordinators from primary schools (70.6%) and from rural areas and small towns (56.8%). The respondents were predominantly women (78.8%) between 50 and 59 years of age (35.6%). Of the schools represented in the study, 36.4% had been part of the HPS network for up to 5 years (182 schools), and 19.0% for 6 to 10 years (95 schools). More than one-fifth of the schools represented in the survey (22.6%, *n* = 113 schools) hold the National Health Promoting School Certificate (Table 2).

**Table 2:** Characteristics of the study sample (*N* = 500)

Specification	Category	Number	Percentage
Duration of the school’s membership in the HPS network	1–5	182	36.4
	6–10	95	19.0
	11–15	84	16.8
	≥ 16	79	15.8
	No response	60	12.0
National HPS Certificate	Yes	113	22.6
	No	315	63.0
	No response	72	14.4
Duration of holding the National HPS Certificate	1–5	41	8.2
	6–10	36	7.2
	11–15	18	3.6
	≥ 16	11	2.2
	No response	394	78.8
School type	Primary school	353	70.6
	Secondary School	80	16.0
	No response	67	13.4
School location	Rural areas and towns (≤ 50,000 inhabitants)	284	56.8
	Medium and large cities (> 200,000 inhabitants)	153	30.6
	No response	63	12.6
Gender of the respondents	Male	37	7.4
	Female	394	78.8
	No response	69	13.8
Age range of the respondents in years	≤ 29	3	0.6
	30–39	65	13.0
	40–49	161	32.2
	50–59	178	35.6
	≥ 60	22	4.4
	No response	71	14.2
Years of professional experience	< 5	12	2.8
	5–15	88	20.6
	16–25	145	33.9
	26–35	129	30.1
	> 35	54	12.6
TOTAL		500	100.0

Percentages are calculated based on the total sample (*N* = 500)

### Level of HPS implementation

With regard to the HPS *Overall implementation* level, a mean value of 3.05 (*SD* = 0.62) was observed across all seven dimensions. As shown in Table 2, the highest implementation scores were found for the dimensions of *Integration* (*M* = 3.53, *SD* = 0.74) *Adaptation* (*M* = 3.40, *SD* = 0.88) and *Programme differentiation* (*M* = 3.35, *SD* = 0.92). In contrast, the dimensions of *Adherence* (*M* = 2.09, *SD* = 0.70) and *Participant responsiveness* (*M* = 2.71, *SD* = 0.84) received the lowest scores (Table 3).

**Table 3:** Level of HPS implementation for subscales and overall implementation

Dimension	*n*	*M* (*SD*)
1. Adherence	476	2.09 (0.70)
2. Dose	500	3.18 (0.82)
3. Participant responsiveness	500	2.71 (0.84)
4. Quality of delivery	491	3.16 (0.79)
5. Integration	485	3.53 (0.74)
6. Programme differentiation	482	3.35 (0.92)
7. Adaptation	482	3.40 (0.88)
***Overall implementation***	458	3.05 (0.62)

Scale: 0–4

*n *sample size, *M  *mean, *SD *= standard deviation

### Differences in HPS implementation by school type

Table 4 shows the differences in the level of HPS implementation for all dimensions between primary and secondary schools. The results show that primary schools scored higher than secondary schools with statistically significant differences for all dimensions except for ‘Adherence’ (*p* < 0.05 to *p* < 0.01). The largest differences with moderate effect sizes were found for ‘*Dose’* (t = 3.17, *p* < 0.01, *d* = 0.39) and *Programme differentiation* (t = 3.01, *p* < 0.01, *d* = 0.37). Differences in *Participant responsiveness*, *Quality of delivery*, *Integration*,* Adaptation* and *Overall implementation* were also statistically significant, and reached small effect sizes (*d* = 0.25–0.36).

**Table 4:** Differences in HPS implementation by school type

Dimension	Primary schools		Secondary schools		t	df	Cohen’s *d*
	*N*	*M* (*SD*)	*N*	*M* (*SD*)			
Adherence	337	2.14 (0.69)	76	2.03 (0.67)	1.20	411	0.15
Dose	335	3.28 (0.74)	80	2.98 (0.82)	3.17**	431	0.39
Participant responsiveness	335	2.80 (0.79)	80	2.53 (0.88)	2.69**	431	0.33
Quality of delivery	335	3.23 (0.72)	80	3.04 (0.82)	2.06*	431	0.25
Integration	335	3.59 (0.68)	80	3.37 (0.76)	2.49**	431	0.31
Programme differentiation	335	3.43 (0.84)	80	3.11 (1.00)	3.01**	431	0.37
Adaptation	335	3.46 (0.82)	80	3.23 (0.98)	2.11*	431	0.26
*Overall implementation*	337	3.10 (0.58)	76	2.88 (0.63)	2.87**	411	0.36

Scale: 0–4

*M  *mean, *SD  *standard deviation, *t  *Student’s t-test, *df  *degrees of freedom, Cohen’s* d*  effect size

*p < 0.05; **p < 0.01

### Differences in HPS implementation by length of HPS network membership

Regarding the membership length in the Polish national HPS network, findings show significant differences on HPS implementation (except for the dimensions *Programme differentiation* and *Adaptation*) in favour of schools belonging to the network (Table 5).

**Table 5:** Differences in HPS implementation by HPS network membership length

Dimension	< 5 years (A)		5–10 years (B)		> 10 years (C)		F (df)	η²	Post-hoc
	*n*	*M* (*SD*)	*n*	*M* (SD)	*n*	*M* (*SD*)			
Adherence	182	1.99 (0.72)	95	2.13 (0.61)	163	2.27 (0.66)	6.48** (2, 415)	0.030	C > A
Dose	182	3.07 (0.84)	95	3.2 (0.87)	163	3.41 (0.59)	7.95*** (2, 437)	0.035	C > A
Participant responsiveness	182	2.62 (0.92)	95	2.77 (0.84)	163	2.90 (0.71)	5.04** (2, 437)	0.023	C > A
Quality of delivery	182	3.05 (0.83)	95	3.20 (0.84)	163	3.37 (0.59)	7.81*** (2, 437)	0.035	C > A
Integration	182	3.47 (0.79)	95	3.46 (0.84)	163	3.68 (0.53)	4.46* (2, 437)	0.020	C > A
Programme differentiation	182	3.29 (0.95)	95	3.41 (0.94)	163	3.46 (0.79)	1.60 (2, 437)	0.007	---
Adaptation	182	3.31 (0.92)	95	3.45 (0.94)	163	3.52 (0.76)	2.55 (2, 437)	0.012	---
*Overall implementation*	182	2.93 (0.68)	95	3.13 (0.54)	163	3.19 (0.49)	8.61*** (2, 415)	0.040	B > A C > A

Scale: 0–4; Oneway ANOVA; Post-hoc Bonferroni; A = up to 5 years of school’s belonging to the HPS network; B = 5–10 years of school’s belonging to the HPS network; C = over 10 years of school’s belonging to the HPS network

*M  *mean, *SD *standard deviation

*p < 0.05; **p < 0.01; ***p < 0.001

Effect size estimates (η²) indicated that the observed effects were small in magnitude. The largest effects were observed for the *Overall implementation* score (η² = 0.040), *Dose* (η² = 0.035) and *Quality of delivery* (η² = 0.035). Post hoc comparisons revealed that schools with the longest membership length in the Network (more than 10 years) achieved significantly higher implementation scores than those with the shortest membership length (> 5 years) in terms of *Adherence*,* Dose*,* Participants’ responsiveness*,* Quality of delivery*,* Integration*, and *Overall implementation*. Although schools that have been part of the HPS network for 5 to 10 years show higher implementation scores than those with shorter membership periods, the differences were not statistically significant.

### Comparison of implementation scores between certified and non-certified schools

As shown in Table 6 schools holding the National Certificate scored significantly higher across all HPS implementation dimensions compared to schools without the certificate (*p* < 0.05 to *p* < 0.001). Statistically significant differences were observed in terms of *Adherence*,* Dose*,* Participant responsiveness*,* Quality of delivery*,* Integration*,* Programme differentiation*,* Adaptation* and *Overall implementation*. However, the sizes of these effects were always small (*d* = 0.23–0.41).

**Table 6:** The differences in HPS implementation dimensions by National Certificate

Dimension	Schools with National Certificate of HPS		Schools without National Certificate of HPS		t	df	Cohen’s *d*
	*n*	*M* (*SD*)	*n*	*M* (*SD*)			
Adherence	109	2.26 (0.66)	302	2.07 (0.69)	-2.52*	409	0.28
Dose	113	3.46 (0.57)	315	3.16 (0.79)	-4.30***	273,70^a^	0.41
Participant responsiveness	113	2.91 (0.71)	315	2.71 (0.83)	-2.24*	426	0.25
Quality of delivery	113	3.38 (0.61)	315	3.15 (0.76)	-3.26**	243,78^a^	0.32
Integration	113	3.68 (0.52)	315	3.52 (0.72)	-2.46*	272,35^a^	0.23
Programme differentiation	113	3.61 (0.63)	315	3.31 (0.92)	-3.75***	288,45^a^	0.35
Adaptation	113	3.61 (0.66)	315	3.36 (0.89)	-3.03**	266,69^a^	0.29
*Overall implementation*	109	3.21 (0.47)	302	3.01 (0.63)	-3.51***	249,99^a^	0.35

Scale: 0–4

*M  *mean, *SD  *standard deviation, *t  *Student’s t-test, *df  *degrees of freedom, Cohen’s* d*  effect size

*p < 0.05; **p < 0.01; ***p < 0.001

## Discussion

This cross-sectional study provides an in-depth analysis of the implementation of the Health Promoting Schools (HPS) approach among schools belonging to the Polish HPS network. Overall, the findings indicate a moderate-to-high level of HPS implementation, with a mean value of 3.05 on a 0–4 scale. However, this aggregate figure masks considerable variability across dimensions. While schools scored highly on Integration and Adaptation, the notably lower scores for Adherence (*M* = 2.09) and Participant responsiveness (*M* = 2.71) suggest that implementation is uneven across dimensions. Rather than full, balanced implementation of the HPS approach, the pattern indicates selective implementation characterised by strong contextual adaptation and systemic embedding, but weaker fidelity to core programme components and limited active engagement of the target population. This aligns with Vennegoor et al.‘s [42] observation that high adaptation scores may coexist with lower adherence, reflecting a pragmatic approach to implementation that prioritises feasibility and local fit over strict programme fidelity.”

### Patterns of implementation across HPS dimensions

The highest implementation scores were observed for *Integration* and *Adaptation*, indicating that health promotion activities are well-embedded in school systems and adjusted to local needs. This aligns with contemporary perspectives that view HPS as a flexible, context-sensitive approach rather than a rigid programme model. These findings support previous research emphasising the importance of adaptability and systemic integration for sustaining HP initiatives in schools [18, 21, 42].

Conversely, lower scores were found for *Adherence* and *Participant responsiveness*. This pattern suggests that while schools actively adapt and integrate health promotion, strict adherence to the HPS framework may be less pronounced. Results of this study show strong convergence with findings from the Dutch cross-sectional study by Vennegoor et al. [42], where surveyed schools (*n* = 535) demonstrated the highest levels of implementation in the dimensions of *Integration* and *Adaptation*, and the lowest in *Adherence* and *Participant responsiveness*. This also reflects an inherent tension identified in the literature between implementation fidelity and contextual adaptation. In line with recent implementation science, reduced *Adherence* does not necessarily indicate poorer implementation quality, but may instead reflect purposeful contextualisation aimed at improving relevance and feasibility within specific school contexts [31, 32, 48]. This observation also aligns with trends identified in various other nations. Research conducted across 24 member countries of the Schools for Health in Europe (SHE) network [49] revealed significant variance among the core components, suggesting that the majority of countries do not prioritize all core components concurrently. It is likely that countries will initiate their efforts with the core components that are most compatible with their specific national contexts. It is therefore advisable for countries to pursue a step-by-step strategy when implementing the core components, which on the one hand takes into account the specific characteristics of the country and the school, but over time leads to a holistic approach [26].

More importantly, the dimensional profile reveals a pattern of selective implementation: Schools have successfully adapted the HPS approach to their contexts and integrated it into organisational structures, but demonstrate weaker adherence to core programme components and limited participant engagement. This raises questions about whether the HPS approach is being implemented in its intended comprehensive form, or whether schools are gravitating towards elements that are more feasible to implement (e.g., contextual adaptation and systemic integration), while neglecting more demanding components (e.g., active student and community involvement). If the components that are theorised to drive the HPS approach’s effectiveness are not delivered as intended, the programme’s impact on health and educational outcomes may be limited, even where overall implementation scores appear favourable [21]. Future research within the Polish HPS network should therefore examine not only the level but the completeness of implementation, and whether the selective pattern observed here is associated with variation in health and educational outcomes across schools.

### Differences between primary and secondary schools

The results demonstrate that primary schools achieve higher implementation levels across most dimensions compared to secondary schools. These differences were particularly evident in *Dose*,* Programme differentiation* and *Overall implementation*. Similar trends have been reported in earlier studies, suggesting that primary schools may have more favourable organisational conditions for whole-school approaches, such as closer teacher–student relationships, greater curricular flexibility, and stronger parental involvement. Moreover, the level of engagement exhibited by parents varies between the two distinct categories of educational institutions [50, 51]. In the initial stages of a child’s education, parents appear to be more involved in the various actions taken by schools [51, 52]. This involvement encompasses not only cognitive growth and academic performance in school, but also their physical health and overall well-being [53–55], which consequently may affect the HPS implementation results. For instance, a German study reported significantly lower implementation of HPS capacity building and concrete actions for secondary schools which indicates the necessity for customised strategies tailored to this school type [10]. In addition, the Dutch findings also demonstrated considerably higher levels of implementation in primary schools than in secondary schools across the majority of dimensions [42]. This finding suggests that organisational characteristics common to primary education – such as smaller size, a curricula focused more on whole-child development, and stronger stakeholder engagement – facilitate the whole-school approach. In contrast, secondary schools often face barriers, including subject fragmentation, higher academic pressures and more limited opportunities for cross-curricular collaboration, which may limit the potential of comprehensive HP initiatives. Notably, no significant differences were observed for *Adherence*, which suggests that differences in implementation are more closely related to intensity, quality and engagement rather than formal compliance with HPS principles. Achieving a balance between maintaining programme fidelity and making necessary adaptations, while also ensuring effective integration of the programme, is crucial for successful implementation and the realization of its intended outcomes [45]. These observations align with findings from other research that emphasize the necessity for a deeper integration of health promotion strategies into the school curriculum [47, 56].

### Length in the network and implementation score

This study revealed differences in implementation by length of HPS network membership, suggesting the role of time and experience. Schools with more than ten years of membership achieved higher scores in most implementation dimensions compared to newer members. Concurrently, although the length of the HPS network is significant [18, 33], this fact alone is unlikely to ensure the effective implementation of the assumptions of the holistic approach to health in schools, particularly in the present study where the effect sizes were negligible. In this context, it is hypothesised that the duration of HPS membership serves as a proxy variable, with other contextual factors potentially exerting a more pronounced influence in specific educational institutions and/or during particular periods. To illustrate this point, it is important to consider that the duration of the network may be less significant than the occurrences within the network and schools. It is evident that further research is required to identify the various forms of support that are offered, including but not limited to: Financial assistance, the involvement of teachers in the HPS implementation, assistance from parents, external partners, experts, and others. Additionally, it is crucial to determine which approaches are suitable for specific schools and under what circumstances, with the aim of enhancing the implementation process and reducing variability in outcomes [5, 31, 45, 57].

### Implementation scores in certified versus non-certified schools

Schools holding the National HPS Certificate scored significantly higher than non-certified schools across all implementation dimensions. The largest differences were observed for Dose (*d* = 0.41) and Programme differentiation (*d* = 0.35), with smaller but consistent differences for other dimensions (*d* = 0.23–0.41).

This pattern is expected given the structure of the Polish certification system. National certification requires schools to document at least three years of sustained health promotion activities, demonstrate systematic planning and evaluation, and provide evidence of good practice dissemination [40]. Schools meeting these criteria would, by design, be expected to score higher on measures of implementation quality. Consequently, the observed differences should be interpreted as indicating alignment between the certification criteria and the implementation dimensions measured by the questionnaire, rather than as evidence that certification causes improved implementation. The findings suggest that the certification process effectively distinguishes schools with stronger implementation profiles from those with weaker profiles.

## Limitations

Several limitations of this study should be acknowledged when interpreting the findings. First, the cross-sectional design does not allow for causal inferences regarding the relationships between school characteristics and the level of HPS implementation. Consequently, observed differences between school types, certification status, or membership length in the network cannot be interpreted as cause–effect relationships. Second, data were collected exclusively through self-report questionnaires completed by school HP coordinators. Regarding the comparison between certified and non-certified schools, observed differences reflect the alignment between certification criteria and questionnaire dimensions rather than an independent effect of certification. Longitudinal designs would be required to examine whether the certification process has additional reinforcing effects on implementation. Although the Polish version of the HPS implementation questionnaire followed a standardized process of translation and adaptation and demonstrated high internal consistency, formal psychometric validation (e.g., confirmatory factor analysis) has yet to be conducted. This limits our ability to confirm that the original factor structure holds in the Polish context, and future studies should address this gap.

Although coordinators are well positioned to assess implementation processes, reliance on a single informant may introduce reporting and social desirability biases, potentially leading to an overestimation of implementation levels. The absence of triangulation with data from other stakeholders (such as teachers, students, parents) or objective measures (such as document analysis, observations) limits the comprehensiveness of the assessment. Third, the study sample consisted solely of schools belonging to the Polish HPS network. As a result, the findings cannot be generalised to all schools in Poland, particularly those not engaged in the HPS initiative. As shown in the study by Dadaczynski et al. [57], schools that belong to a federal programme did only partially differ in their HPS implementation compared to those who do not during the COVID pandemic.

Due to a lack of data on the total population of the HPS network, it is not possible to assess its representativeness, for example, with regard to school type. However, compared to the overall sample of all Polish schools, our sample included slightly more primary schools. The voluntary nature may have introduced selection bias, with more engaged or better-resourced schools potentially overrepresented. Consequently, the reported implementation levels may be higher than those of the HPS network as a whole.

While the study examined several important school-related characteristics, a number of potentially confounding variables were not measured and could not be controlled for. These include: School size and resources (which may affect the capacity to implement comprehensive health programmes [10, 58]); the socioeconomic profile of the school’s catchment area (which may shape both health needs and available community support); school leadership style and principal health literacy (which have been identified as key determinants of HPS implementation [9, 11]); and the availability of external partners such as local government, health services, or NGOs. Because these factors may co-vary with school type, certification status, or network membership length, the observed group differences should be interpreted with caution and may not reflect the independent effect of the variables under study. Hence, future studies should include a broader set of contextual covariates that allow for statistical control of confounding.

Finally, this study assessed implementation processes and did not measure health or educational outcomes among students or staff. Consequently, we cannot determine whether the observed implementation levels translate into improved health, well-being, or academic performance. Future research should link implementation data with outcome measures to evaluate the real-world impact of the HPS approach. 

### Conclusions and implications for research and practice

The findings of the present study confirm the value of multidimensional implementation frameworks that move beyond linear notions of programme fidelity. Sustained participation in the HPS network was associated with higher implementation scores, suggesting that long-term engagement supports capacity building and programme consolidation. Schools holding the National HPS Certificate scored higher across all implementation dimensions, a pattern consistent with the rigorous requirements of the certification process. At the system level, further development of monitoring, evaluation and certification mechanisms remains crucial to ensure that HPS initiatives respond to locally relevant health priorities rather than symbolic compliance. Sustained investment in professional development, participatory leadership and network-level capacity building will be essential to support democratic school cultures and to integrate emerging priorities such as environmental sustainability into whole-school health promotion. Given the implementation levels observed among network schools, efforts to expand the HPS programme to additional schools across Poland appear warranted. Strategies to support newly joining schools, drawing on the experiences of long-standing network members, could facilitate broader adoption of the HPS approach. 

## Data Availability

The translated questionnaire and the datasets used in this study are available upon request to the corresponding author.

## Abbreviations

HPS: Health Promoting School
WHO: World Health Organization
ORE: Centre of Education Development
HP: Health Promotion
SHE: Schools for Health in Europe
